# The levels of vascular endothelial cells and endothelium-dependent vasomotor cytokines in children with essential hypertension: a case-control study

**DOI:** 10.3389/fped.2026.1855686

**Published:** 2026-06-18

**Authors:** Jingjing Ma, Yaxi Cui, Yao Lin, Yanyan Liu, Yang Liu, Lin Shi

**Affiliations:** Department of Cardiology, Capital Center for Children’s Health, Capital Medical University, Capital Institute of Pediatrics, Beijing, China

**Keywords:** child, endothelial cells, endothelial function, endothelium-dependent vasomotor cytokines, essential hypertension

## Abstract

**Background:**

Circulating endothelial cells (CECs), endothelial progenitor cells (EPCs) and endothelium-dependent vasomotor cytokines are important in maintaining endothelial function stability. However, these alterations have not been documented in childhood essential hypertension. This study aim to investigate the levels of CECs, EPCs and endothelium-dependent vasomotor cytokines in children with essential hypertension.

**Methods:**

A total of 101 children with essential hypertension and 30 healthy children were recruited as hypertension (HTN) group and control group, respectively. Peripheral CEC and EPC counts, nitrite (NO_2_^−^), endothelin-1 (ET-1), thromboxane B2 (TXB_2_) and 6-keto-prostaglandin F1a (6-keto-PGF1a) were detected and compared between the two groups. Correlations between 24 h mean arterial pressure and endothelium-dependent vasomotor cytokines was analyzed.

**Results:**

The level of CECs in the HTN group was significantly higher than that in the control group (*p* < 0.01), while the EPCs were significantly lower (*p* < 0.01). The concentrations of NO_2_^−^ (*p* < 0.01) and 6-keto-PGF1a (*p* < 0.0001) in the HTN group were significantly lower than those in the control group, while the levels of ET-1 (*p* < 0.0001) and TXB_2_ (*p* < 0.001) were significantly higher. The levels of NO_2_^−^ and 6-keto-PGF1a demonstrated a negative correlation with 24-hour mean arterial pressure (*r* = −0.31, *p* < 0.01; *r* = −0.44, *p* < 0.001), whereas TXB_2_ and ET-1 levels exhibited a positive correlation (*r* = 0.24, *p* < 0.05; *r* = 0.31, *p* < 0.01).

**Conclusion:**

CECs, EPCs and endothelium-dependent vasomotor cytokines were altered in pediatric essential hypertension, suggesting that endothelial dysfunction may play a role in its pathogenesis.

## Introduction

In recent years, the prevalence of pediatric essential hypertension has been on the rise. A study in China showed that the prevalence of hypertension among children increased from 8.5% to 19.2% over the last two decades (1). Some studies suggest that endothelial cell damage is the initiating factor of the “endothelial-hypertensive-cardiovascular event chain” in the progression of adult hypertension (2, 3). Peripheral circulating endothelial cells (CECs) and circulating endothelial progenitor cells (EPCs) are well-recognized vascular endothelial functional markers. Previous studies have shown that in adults hypertension, endothelial injury is characterized by increased CEC counts and impaired EPC regenerative capacity which relies on the migration of EPCs to injured endothelium and their differentiation into mature endothelial cells *in situ* (4). Beyond cellular alterations in endothelial populations, vascular endothelial function is also tightly regulated by a panel of vasoactive cytokines secreted by endothelial cells. In addition, the endothelium can synthesize and release various vasoactive cytokines, termed endothelium-dependent vasomotor cytokines, including vasodilatory factors such as nitric oxide (NO), prostacyclin (PGI_2_) and endothelium-derived hyperpolarizing factor (EDHF), as well as vasoconstrictive factors such as thromboxane (TXA_2_) and endothelin-1 (ET-1) (5). Imbalanced secretion and regulation of these vasomotor cytokines may lead to vascular pathophysiological changes. Although endothelial dysfunction has been extensively investigated in adult hypertension, these findings cannot be directly extrapolated to children. Pediatric essential hypertension differs from adult hypertension in terms of disease duration, vascular remodeling, developmental status, and early target organ damage. Therefore, independent investigations in pediatric populations are necessary to determine whether similar endothelial injury and vasomotor dysregulation occur in the early stages of hypertension. Furthermore, early identification of endothelial dysfunction in children with hypertension may improve the understanding of the pathophysiological mechanisms underlying early vascular injury and provide potential diagnostic and prognostic evidence for cardiovascular risk assessment in pediatric hypertension. However, data regarding endothelial cells and vasomotor cytokines in childhood essential hypertension remain limited. Therefore, this case-control study aimed to evaluate the levels of CECs, EPCs, and vasomotor cytokines in children with essential hypertension and to explore their potential associations with endothelial injury and vasomotor dysfunction. The findings may provide novel potential biomarkers for early diagnosis, risk stratification, and clinical intervention for pediatric hypertension.

## Methods

### Ethics statement

The study protocol was approved by the Ethics Committee of the Capital Institute of Pediatrics, Beijing (No. SHERLL2025030), in accordance with the principles outlined in the Declaration of Helsinki. Written informed consent was obtained from all participants or the guardians of minor subjects.

### Study subjects

A total of 101 children aged 9–16 years who were diagnosed with essential hypertension in our hospital from August 2017 to January 2019 were enrolled as the hypertension (HTN) group. The diagnosis and classification of pediatric hypertension followed the 2024 Chinese Guidelines for the Prevention and Treatment of Hypertension (6). For children aged 3–15 years, hypertension was diagnosed strictly in accordance with the above guidelines. Hypertension was defined as systolic blood pressure (SBP) and/or diastolic blood pressure (DBP) reaching or exceeding the 95th age-, sex- and height-specific blood pressure percentile, confirmed by no fewer than three standardized clinical measurements performed by specialized pediatric cardiologists. A single blood pressure measurement ≥99th percentile plus 5 mmHg was also defined as definitive hypertension. For adolescents aged 16–17 years, whose physical development and sexual maturation are comparable to those of adults, adult hypertension diagnostic criteria were directly adopted: hypertension was defined as SBP ≥ 140 mmHg and/or DBP ≥ 90 mmHg. For children and adolescents aged 3–15 years, normal blood pressure is defined as values below the 90th percentile corresponding to age, sex and height. For adolescents aged 16 years and older, normal blood pressure is less than 120/80 mmHg. All blood measurements were performed by well-trained investigators using a validated automated sphygmomanometer under standardized conditions. After a minimum 5 min resting period, children remained in a comfortable seated position with their backs supported and feet flat on the floor. The right arm was maintained at heart level throughout the measurement. Appropriate cuff size was selected based on the upper arm circumference, ensuring that the cuff bladder width covered 40%–50% of the arm circumference and the bladder length covered 80%–100% of the arm circumference. Three BP readings were obtained at 1–2 min intervals, and the mean value of the last two readings was used for subsequent statistical analyses. The exclusion criteria for this study were secondary hypertension caused by kidney diseases, vascular diseases, endocrine disorders, central nervous system diseases, or medication exposure. Thirty age- and sex-matched healthy children underwent healthy physical examination with normal blood pressure in our hospital during the same period were recruited as the control group. Healthy controls were defined as children without a history of hypertension, cardiovascular diseases, renal diseases, endocrine disorders, acute or chronic inflammatory diseases, or recent infections. All control participants completed a comprehensive routine examination, including medical history assessment, physical examination, and standard laboratory tests. No subclinical diseases or abnormal laboratory findings were detected at enrollment.

Sample size justification: A power analysis was performed using G*Power 3.1 software to determine the appropriate sample size for this case—control study. Based on previous studies regarding endothelial markers in hypertension, we assumed a medium effect size (*d* = 0.5), a significance level (*α* = 0.05), and a statistical power (1−*β* = 0.85). The calculated minimum sample size required for each group was 28 participants. We ultimately recruited 101 children in the hypertension group and 30 children in the control group, which adequately met and exceeded the calculated minimum sample size requirement.

### Samples

Four milliliters of fasting venous blood were obtained in the early morning. Of the total volume, 2 mL was drawn into a heparin anticoagulant tube for flow cytometric analysis, and the remaining 2 mL was transferred to an EDTA anticoagulant tube and centrifugation at 4 °C at 3,000 rpm for 10 min. The supernatant was stored at −70 °C for detecting NO_2_^−^, ET-1, TXB_2_, and 6-keto-PGF1a.

### Flow cytometry detection of CECs and EPCs

Peripheral venous blood was collected into heparin anticoagulant tubes. Red blood cells were lysed using RBC lysis buffer (Invitrogen, #00-4333-57) for 10 min at room temperature in the dark. Cells were then washed and resuspended in Hank's balanced saline solution supplemented with 2% FBS. Single-cell suspensions were stained with the following anti-human antibodies: CD146-APC (clone P1H12, eBioscience), CD45-PerCP-Cy5.5 (clone J33, Beckman Coulter, #IM2650U), CD133-PE (clone TMP4, eBioscience, #17-1338-42), and CD34-FITC (clone 581, Beckman Coulter, #IM1870U). 7-AAD Viability Dye (Beckman Coulter, #A07704) was added immediately before acquisition to discriminate dead cells. Flow cytometry was performed using an LSRFortessa cell analyzer (BD Immunocytometry Systems). Fluorescence minus one (FMO) controls were used for each fluorochrome to define positive gate boundaries. Isotype controls were used as supplementary controls. The gating strategy was as follows: FSC-A vs. SSC-A to exclude debris, FSC-H vs. FSC-A to exclude cell doublets and aggregates, and a viability gate (7-AAD-negative) to select live cells. Cells with bright CD45 expression (leukocytes) were excluded, and CD45-negative/dim cells were selected for further analysis. CECs were identified as CD45⁻/CD34⁺/CD133⁻/CD146⁺, whereas EPCs were identified as CD45⁻/CD34⁺/CD133⁺/CD146⁺ (7). Flow cytometric data were analyzed using Kaluza Analysis 1.0 software (Beckman Coulter).

### Measurement of plasma NO_2_^−^ concentrations

NO has a short half-life in the oxygen-containing aqueous solution, therefore, this study used nitrate reductase to specifically reduce NO_3_^−^ to NO_2_^−^, thereby indirectly reflecting plasma NO levels. Plasma nitric oxide (NO) concentrations were indirectly determined by measuring stable nitric oxide metabolites (nitrite/nitrate, NOx) using the Nitric Oxide (NO) Assay Kit (Catalog No. A012-1, Nanjing Jiancheng Bioengineering Institute, China) according to the manufacturer's protocols and previously published methods based on the Griess reaction assay. Peripheral venous blood samples were collected into anticoagulant tubes and centrifuged to obtain plasma, which was stored at −80 °C before analysis. Prior to detection, nitrate (NO_3_⁻) in the plasma samples was reduced to nitrite (NO_2_⁻) using nitrate reductase. Briefly, 100 μL of plasma sample or standard solution was added to each reaction well and incubated with the reaction reagents at 37 °C for 60 min. After addition of the chromogenic reagents, the mixtures were vortexed, incubated at room temperature, and centrifuged at 3,500–4,000 rpm for 10 min. Absorbance was measured at 550 nm using an Infinite 200 Pro NanoQuant full-wavelength microplate reader (Tecan, Männedorf, Austria), with distilled water used as the blank control. All samples were analyzed in duplicate, and NO concentrations were calculated according to the standard calibration curve provided by the kit. All experimental operations were performed in accordance with the manufacturer's protocol. Standard calibration curves were generated using serial standard solutions supplied with the kit before each assay. The intra-assay and inter-assay coefficients of variation were both less than 10% according to the manufacturer's specifications.

### Measurement of plasma ET-1, TXB_2_, PGI_2_ concentrations

All subjects underwent early morning fasting venous blood collection (2 mL) after admission. Blood samples were placed in EDTA anticoagulant tubes and centrifuged at 3,000 r/min for 10 min at 4 °C. The separated supernatant was stored at −70 °C, and repeated freeze–thaw cycles were strictly avoided. After all sample collection was completed, plasma levels of ET-1, TXB_2_ and 6-keto-PGF1a were uniformly measured by enzyme-linked immunosorbent assay (ELISA), with all samples tested in duplicate. The assays were performed strictly according to the instructions of commercial kits: Endothelin-1 Assay Kit (Catalog No. H204-1-1, Nanjing Jiancheng Bioengineering Institute, China), Thromboxane B2 Assay Kit (Catalog No. H173-1-2, Nanjing Jiancheng Bioengineering Institute, China), and 6-keto-prostaglandin F1a Assay Kit (Catalog No. H204-1-2, Nanjing Jiancheng Bioengineering Institute, China). Absorbance was read at 450 nm using an Infinite 200 Pro NanoQuant microplate reader (Tecan, Männedorf, Austria), and plasma concentrations were calculated subsequently. Standard calibration curves were established for each biomarker assay using kit-provided standards. All samples and standards were measured in duplicate, and the average values were used for analysis. According to the manufacturer's instructions, the intra-assay and inter-assay coefficients of variation for all ELISA kits were lower than 10%.

### Statistical analysis

Normality of all variables was assessed using the Shapiro–Wilk test. Normally distributed data including plasma NO_2_⁻, TXB_2_ and 6-keto-PGF1a were presented as mean ± standard deviation and compared by the independent-samples *t*-test. Non-normally distributed data, such as CECs and EPCs counts, were expressed as median (Q1, Q3) and analyzed using the Mann–Whitney *U* test. The association of NO_2_^−^, ET-1, TXB_2_, and 6-keto-PGF1a with mean arterial pressure was analyzed by the Spearman correlation. *P* < 0.05 was considered statistically significant. Statistical analyses were performed using the SPSS 30.0 (SPSS, Chicago, IL, USA).

## Results

### Demographics and blood pressure level

There was no significant difference between the two groups in terms of age, gender, BMI, blood lipid, blood glucose and hepatic and renal function (*p* > 0.05). The 24 h average systolic blood pressure and average diastolic blood pressure in the hypertension group were significantly higher than those in the control group (127.55 ± 10.82 vs. 105.00 ± 7.94 mmHg, *t* = 8.834, *p* < 0.01; 71.19 ± 6.31 vs. 66.13 ± 9.77 mmHg, *t* = 2.172, *p* < 0.05) (Table 1).

**Table 1:** Demographics and blood pressure levels between the two groups.

Variable	Hypertension group (*n* = 101)	Control group (*n* = 30)	*t*/*χ*^2^ value	*p* value
Age	12.70 ± 1.65	11.78 ± 2.19	1.770	0.083
Gender (male/female)	59:42	14:16	1.294	0.255
BMI (kg/m^2^)	23.74 ± 3.40	22.28 ± 1.94	2.001	0.051
24 h average systolic blood pressure (mmHg)	127.55 ± 10.82	105.00 ± 7.94	8.834	<0.01
24 h average diastolic blood pressure (mmHg)	71.19 ± 6.3	66.13 ± 9.77	2.172	0.037
Triglyceride (mmol/L)	1.19 ± 0.51	1.03 ± 0.79	0.866	0.390
Cholesterol (mmol/L)	3.89 ± 0.72	3.63 ± 0.47	1.633	0.109
High density lipoprotein (mmol/L)	1.20 ± 0.16	1.35 ± 0.32	−1.963	0.059
Low density lipoprotein (mmol/L)	2.14 ± 0.50	1.95 ± 0.48	1.395	0.169
Blood glucose (mmol/L)	4.43 ± 0.44	4.66 ± 0.40	−1.925	0.060
Alanine aminotransferase (U/L)	17.17 ± 6.09	15.22 ± 5.17	1.238	0.221
Aspartate aminotransferase (U/L)	21.69 ± 7.43	20.11 ± 4.74	0.946	0.348
Serum creatinine (µmol/L)	52.09 ± 11.26	47.50 ± 9.63	1.574	0.122
Blood urea nitrogen (mmol/L)	4.50 ± 0.71	4.74 ± 1.08	−0.924	0.362

Data are expressed as mean ± SD for normally distributed variables. Between-group differences were compared using independent-sample *t*-test. *P* < 0.05 indicated statistical significance.

**Figure 1:**
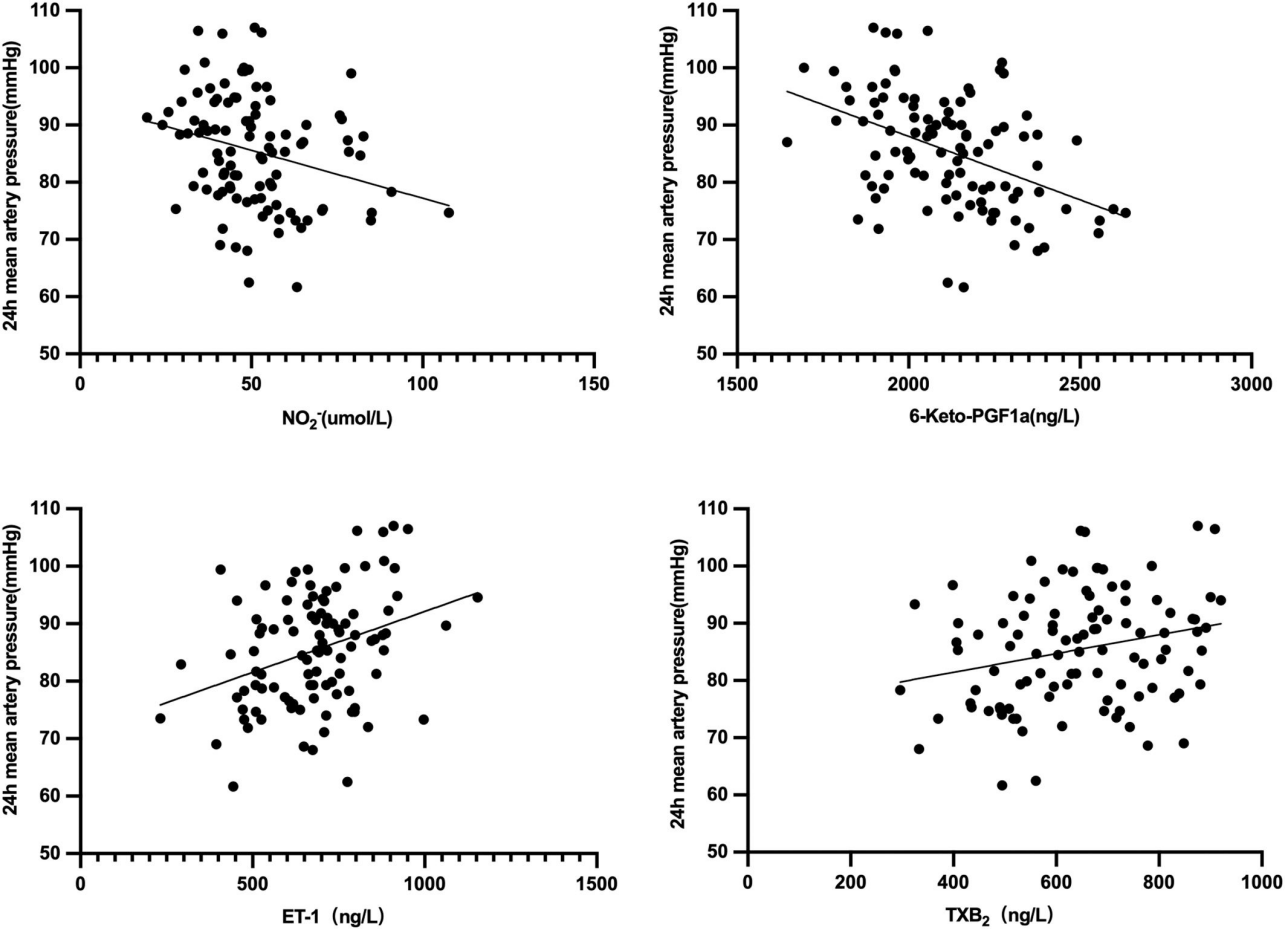
Correlation between NO_2_^−^, ET-1, TXB_2_, and 6-keto-PGF1a and 24 h mean blood pressure (mmHg) in hypertension group. Spearman correlation analysis was used to examine the relationships between 24 h mean blood pressure parameters and endothelial function markers in hypertension group. *P* < 0.05 was regarded as statistically significant.

The median CECs count in the hypertension group was significantly higher than that of the control group (*Z* = −7.27, *p* < 0.01). The median EPCs count in the hypertension group was significantly lower than that of the control group (*Z* = −2.38, *p* < 0.05) (Table 2). Children with hypertension showed significantly lower NO_2_^−^ and 6-keto-PGF1a concentrations, and higher ET-1 and TXB_2_ concentrations compared to controls (all *p* < 0.01; Table 3). There was a negative correlation of the NO_2_^−^, 6-keto-PGF1a concentrations (*r* = −0.31, *p* < 0.01; *r* = −0.44, *p* < 0.001), and a positive correlation of the TXB_2_ and ET-1 concentrations (*r* = 0.24, *p* < 0.05; *r* = 0.31, *p* < 0.01) with the 24 h mean arterial pressure (Figure 1).

**Table 2:** Comparison of peripheral blood CECs and EPCs in the hypertension group and control group.

Variable	Hypertension group (*n* = 101)	Control group (*n* = 30)	*Z* value	*p* value
CECs (cells/μL)	66.0 (53.0, 93.0)	21.0 (14.0, 25.3)	−7.27	<0.01
EPCs (cells/μL)	12.0 (5.0, 27.0)	17.5 (13.8, 30.0)	−2.38	<0.05

Data are expressed as median (IQR) for non-normally distributed variables. Between-group differences were compared using Mann–Whitney *U* test. *P* < 0.05 indicated statistical significance.

**Table 3:** Comparison of NO_2_^−^, ET-1, TXB_2_, and 6-keto-PGF1a in the hypertension group and the control group.

Variable	Hypertension group (*n* = 101)	Control group (*n* = 30)	*t*/*χ*^2^ value	*p* value
NO_2_^−^ (µmol/L)	51.02 ± 15.67	65.23 ± 18.55	−3.045	<0.01
6-keto-PGF1a (ng/L)	2,118.15 ± 196.75	2,432.14 ± 238.39	−5.298	<0.0001
ET-1 (ng/L)	682.50 ± 158.75	422.18 ± 95.31	6.997	<0.0001
TXB_2_ (ng/L)	643.13 ± 149.89	509.68 ± 117.79	3.534	<0.001

Data are expressed as mean ± SD for normally distributed variables. Between-group differences were compared using independent-sample *t*-test. *P* < 0.05 indicated statistical significance.

## Discussion

This study investigated peripheral CECs, EPCs and endothelial-dependent vasomotor cytokines in children and adolescents with essential hypertension. To our knowledge, this is among the first studies to simultaneously evaluate endothelial injury markers and vascular repair markers. We found that the level of CECs in the HTN group was significantly higher than that in the control group, while the EPCs were significantly lower. The concentrations of NO_2_^−^ and 6-keto-PGF1a in the HTN group were significantly lower than those in the control group, while the concentrations of ET-1 and TXB_2_ were significantly higher.

The mechanisms that regulate blood pressure are numerous and complex. Circulating endothelial cells are mature cells shed from blood vessels with a very low peripheral counts under normal physiological conditions. In various cardiovascular diseases, the elevated CECs are considered the direct biomarker of vascular damage. Some studies have found that the count of CECs in adults with hypertension is higher than that of healthy ones, suggesting that endothelial cells are damaged in adults with hypertension (8–10). In this study, the peripheral CECs level in children with essential hypertension was significantly higher than that of healthy children, suggesting direct endothelial cell damage in children with essential hypertension. Increased CECs counts may result from in the enhanced tension of the blood vessel wall and the injured endothelial cells detachment to peripheral blood in the situation of long-term high blood pressure.

EPCs, isolated both from blood and bone marrow, are being widely studied as potential biomarkers for vascular injury and vascular repair. Furthermore, evidence suggest that they can reverse endothelial dysfunction or the vascular damages.

Imanishi et al. (11) found that the blood EPCs counts in essential hypertension rats were reduced and the function was impaired. Lan et al. (12) found that the blood EPCs counts in adult patients with hypertension were reduced, and the ability of EPCs to proliferate, migrate and adhere was declined. In this study, the EPCs count of children with hypertension was lower than that of healthy children, suggesting impaired vascular repair ability of children with essential hypertension. The possible mechanisms may be associated with inhibited EPCs differentiation by renin–angiotensin system (RAS) which is dominant pathogenesis of hypertension. In addition to cellular markers of endothelial injury and repair, we observed significant alterations in endothelial-dependent vasoactive factors. Specifically, NO_2_^−^ and 6-keto-PGF1a concentrations were decreased, whereas ET-1 and TXB_2_ concentrations were increased in the hypertension group. These findings collectively suggest impaired endothelial-dependent vasodilation and enhanced vasoconstrictive activity in pediatric essential hypertension, further supporting the presence of endothelial dysfunction. NO is an important endothelial-derived vasodilator that regulates vascular tone and inhibits vascular smooth muscle contraction. Reduced NO bioavailability has been widely recognized as a hallmark of endothelial dysfunction in hypertension (13–16). In our study, decreased plasma NO_2_^−^ concentrations in hypertensive children suggested impaired endothelial-dependent relaxation function, which may contribute to increased peripheral vascular resistance and elevated blood pressure.

PGI_2_ is another important endothelial-derived vasodilatory and antiplatelet factor, whereas TXA_2_ promotes vasoconstriction and platelet aggregation. The imbalance between PGI_2_ and TXA_2_ may reflect disrupted vascular homeostasis in pediatric hypertension. Due to the instability and difficult measurement of PGI_2_, it is usually assessed by detecting its metabolite 6-keto-PGF1a (17). We observed reduced 6-keto-PGF1a concentrations and elevated TXB_2_ concentrations in hypertensive children, indicating a shift toward vasoconstrictive and prothrombotic endothelial activity.

ET-1, produced by vascular endothelial cells, is among the most potent endogenous vasoconstrictors. Elevated ET-1 concentrations in hypertensive children further support the presence of enhanced endothelial-dependent vasoconstrictive function and vascular dysfunction in pediatric hypertension (18, 19).

Importantly, correlation analysis demonstrated that NO_2_^−^ and 6-keto-PGF1a concentrations were negatively correlated with 24 h mean arterial pressure, whereas ET-1 and TXB_2_ concentrations were positively correlated with blood pressure levels. These findings suggest that endothelial dysfunction is closely related to blood pressure severity in pediatric essential hypertension. The observed correlations further support the potential role of endothelial biomarkers in reflecting disease activity and vascular impairment.

From a clinical perspective, these biomarkers may have potential value in the early identification of endothelial dysfunction and vascular injury in children with hypertension. Combined assessment of CECs, EPCs, and endothelial-dependent vasoactive factors may help improve risk stratification and provide additional information regarding disease progression and therapeutic monitoring. However, their clinical utility requires further validation in large prospective studies.

### Limitations

This study has some limitations. This is a single center study without follow-up data. Further multicenter prospective study should be conducted to observe the long-term changes of these factors, the potent role in pathogenesis, and predictive value for therapy efficacy.

## Conclusion

In conclusion, our findings indicate that pediatric essential hypertension is associated with endothelial injury and impaired vascular repair capacity, accompanied by endothelium-dependent vasomotor dysfunction. These identified biomarkers (CECs, EPCs, NO_2_⁻, 6-keto-PGF1a, ET-1, TXB_2_) may serve as potential clinical indicators for early assessment of vascular damage and risk stratification in children with essential hypertension.

## Data Availability

The raw data supporting the conclusions of this article will be made available by the authors, without undue reservation.
